# Ultrasonic hemodynamic changes of superficial temporal artery graft in different angiogenesis outcomes of Moyamoya disease patients treated with combined revascularization surgery

**DOI:** 10.3389/fneur.2023.1115343

**Published:** 2023-02-16

**Authors:** Siyuan Chen, Baoping Wang, Yunyu Wen, Zhibin Wang, Tinghan Long, Junda Chen, Guozhong Zhang, Mingzhou Li, Shichao Zhang, Jun Pan, Wenfeng Feng, Songtao Qi, Gang Wang

**Affiliations:** ^1^Department of Neurosurgery, Nanfang Hospital, Southern Medical University, Guangzhou, China; ^2^Department of Ultrasonography, Nanfang Hospital, Southern Medical University, Guangzhou, China

**Keywords:** Moyamoya, ultrasonic, combined bypass surgery, hemodynamics, revascularization, forecast

## Abstract

**Objective:**

Combined bypass is commonly used in adult Moyamoya disease (MMD) for revascularization purposes. The blood flow from the external carotid artery system supplied by the superficial temporal artery (STA), middle meningeal artery (MMA), and deep temporal artery (DTA) can restore the impaired hemodynamics of the ischemic brain. In this study we attempted to evaluate the hemodynamic changes of the STA graft and predict the angiogenesis outcomes in MMD patients after combined bypass surgery by using quantitative ultrasonography.

**Methods:**

We retrospectively studied Moyamoya patients who were treated by combined bypass between September 2017 and June 2021 in our hospital. We quantitatively measured the STA with ultrasound and recorded the blood flow, diameter, pulsatility index (PI) and resistance index (RI) to assess graft development preoperatively and at 1 day, 7 days, 3 months, and 6 months after surgery. All patients received both pre- and post- operative angiography evaluation. Patients were divided into either well- or poorly-angiogenesis groups according to the transdural collateral formation status on angiography at 6 months after surgery (W group or P group). Patients with matshushima grade A or B were divided into W group. Patients with matshushima grade C were divided into P group, indicating a poor angiogenesis development.

**Results:**

A total of 52 patients with 54 operated hemispheres were enrolled, including 25 men and 27 women with an average age of 39 ± 14.3 years. Compared to preoperative values, the average blood flow of an STA graft at day 1 postoperation increased from 16.06 ± 12.47 to 117.47± 73.77 (mL/min), diameter increased from 1.14 ± 0.33 to 1.81 ± 0.30 (mm), PI dropped from 1.77 ± 0.42 to 0.76 ± 0.37, and RI dropped from 1.77 ± 0.42 to 0.50 ± 0.12. According to the Matsushima grade at 6 months after surgery, 30 hemispheres qualified as W group and 24 hemispheres as P group. Statistically significant differences were found between the two groups in diameter (*p* = 0.010) as well as flow (*p* = 0.017) at 3 months post-surgery. Flow also remained significantly different at 6 months after surgery (*p* = 0.014). Based on GEE logistic regression evaluation, the patients with higher levels of flow post-operation were more likely to have poorly-compensated collateral. ROC analysis showed that increased flow of ≥69.5 ml/min (*p* = 0.003; AUC = 0.74) or a 604% (*p* = 0.012; AUC = 0.70) increase at 3 months post-surgery compared with the pre-operative value is the cut-off point which had the highest Youden's index for predicting P group. Furthermore, a diameter at 3 months post-surgery that is ≥0.75 mm (*p* = 0.008; AUC = 0.71) or 52% (*p* =0.021; AUC = 0.68) wider than pre-operation also indicates a high risk of poor indirect collateral formation.

**Conclusions:**

The hemodynamic of the STA graft changed significantly after combined bypass surgery. An increased flow of more than 69.5 ml/min at 3 months was a good predictive factor for poor neoangiogenesis in MMD patients treated with combined bypass surgery.

## 1. Introduction

Moyamoya disease (MMD) is an idiopathic chronic cerebrovascular disorder caused by progressive occlusion of the bilateral terminal internal carotid artery (ICA) and its main branches, accompanied by basal collateral formation. To date, a growing number of studies support the use of combined bypass, which has a significant therapeutic effect, as the preferred option of treatment (1, 2). As a combination of direct and indirect bypass, combined bypass surgery provides better revascularization for the adult MMD population by both an instant increase in blood flow from direct anastomosis and subsequent spontaneous ingrowth of collaterals from the indirect bypass (3, 4). Previous studies have reported that neoangiogenesis and improvement of intracranial perfusion in the surgical area during follow-up leads to varied outcomes after surgery (4, 5). Although the STA graft is located outside the skull, its haemodynamic changes may indicate intracranial compensatory collateral and clinical prognosis (6, 7). Many studies have tracked changes in grafts after surgery; however, there is still a lack evidence to substantiate the notion that combined direct/indirect bypass can provide temporary complementary revascularization (8–10).

Cerebral angiography is the gold standard tool for follow-up after combined revascularization surgery in patients with Moyamoya disease, and the standard grading based on Digital Subtraction Angiography (DSA) angiography of collateral circulation proposed by Matsushima et al. could provide a good assessment of efficacy (11–13). However, the invasiveness and radiation exposure of this program impede regular DSA examination (14–17). In many clinical situations, ultrasonography is the preferred non-invasive method for the evaluation of blood flow (18). Our previous studies have shown that quantitative ultrasonography of STA has a direct role in evaluating long-term revascularization in patients (19). However, its usefulness in the early follow-up of post-operative neovascularization remains unclear. This study aimed to correlate the ultrasound parameters and the haemodynamic changes in the STA of patients with Moyamoya disease after combined revascularization with the angiographic Matsushima grade to explore the role of quantitative ultrasound as a follow-up tool.

## 2. Clinical material and methods

### 2.1. Patient selection

This study was approved by the Institutional Review Board of Nanfang Hospital. Patients newly diagnosed with MMD by cerebral angiography between September 2017 and June 2021 at Nanfang Hospital of Southern Medical University were prospectively registered in the current study. We obtained informed consent from all patients in writing and orally. Inclusion criteria were as follows: (1) clear indications for surgery and patient or family members' signed informed consent agreeing to the surgery; (2) the patient received combined bypass surgery (superficial temporal artery-middle cerebral artery bypass combined with encephalo-dura-myo-synangiosis); (3) ultrasound examination was performed before operation and at 1 day, 7 days, 3 months, and 6 months after operation; and (4) DSA angiography was performed at 6 months after surgery to evaluate the effect of surgery. The exclusion criteria were as follows: (1) the patient refused surgery; (2) the follow-up data (ultrasonography at any time point) was missing.

All patients were evaluated by Modified Rankin scale (mRS) and Suzuki stage according to body function and pre-operative DSA angiography. Modified Rankin scale (mRS): 0, no symptoms; 1, no significant disability, despite symptoms; 2, slight disability; 3, moderate disability; 4, moderately severe disability; 5, severe disability; 6, dead. Suzuki stage: stage 1, Stenosis of the distal end of the ICA; stage 2, Stenosis of main intracranial arteries and the forming of the Moyamoya; stage 3, The ICA was further narrowed or occluded, involving the middle cerebral artery (MCA) and anterior cerebral arteries (ACA), and the Moyamoya were more obvious.; stage 4, The whole Willis circle and even the posterior cerebral artery (PCA) were occluded, and extracranial collateral circulation formed; the Moyamoya began to decrease.; stage 5, Reduction of Moyamoya; stage 6, The ICA and its branches were completely occluded and the moyamoya disappeared; the blood supply to the brain was completely dependent on the collateral circulation of the external carotid artery (ECA) and the vertebral-basilar artery system.

According to the Matsushima grading at 6 months post-operation, patients were grouped into two groups (grade A: collateral compensation filling of greater than two thirds of the cortical surface in the territory; grade B: between one third and two thirds; grade C: less than one third). Patients with Matsushima grade A or B were allocated to the well-compensated collateral group (W group) and patients with Matsushima grade C were allocated to the poorly-compensated collateral group (P group). The angiograms were evaluated by two independent observers, and divergence was adjudicated by a third doctor.

### 2.2. Ultrasonography examination of graft

We performed an ultrasound study using duplex ultrasonography and B-mode to examine the graft using Toshiba Aplio 500 machines at our hospital. Two senior neuroradiologists reviewed and recorded the ultrasonography images independently; neither of them were involved in the surgery and they were blinded to the clinical information. The patient was placed in a supine position to maintain the incident angle of 60° or less between the STA and the Doppler beam. Probing the artery trunk of the STA in front of the tragus, we gradually traced along the trunk to the distal end until the STA entered the skull. Branch vessels were confirmed to be operated upon, and the junction of the intracranial-extracranial segment was selected as the check point. If a double barrel was involved, the check point was changed to a location 3–5 mm proximal to the bifurcation of the frontal and parietal branches of the STA.

The blood flow (ml/min), diameter (mm), pulsatility index (PI), and resistance index (RI) values were calculated automatically by the software and recorded when the measurement was usable. The recorded diameter is the maximum internal diameter of a blood vessel during cardiac contraction. The recorded flow is the average blood flow over a complete cardiac cycle. The RI value reflects the elasticity of the vascular wall and the resistance at the distal end of the blood flow. It equals to (Vs-Vd)/Vs, (Vs: peak systolic flow velocity, Vd: end diastolic flow velocity). Pi value reflects the activity, hardness and the resistance of blood vessel during the whole cardiac cycle. It equals to (Vs-Vd)/Vm (Vm: Space Peak time average velocity, the average value of flow velocity at each point during the whole cardiac cycle).

Ultrasound examination was conducted for patients pre-operatively, and follow-ups were scheduled at 1 day, 7 days, 3 months, and 6 months after surgery. Ultrasonographic data were recorded only according to the examination results, and data acquisition was blinded to the angiographic results.

### 2.3. Brief description of surgical procedure

All patients underwent combined bypass surgery performed by two experienced surgeons using a standard procedure. A question mark flap was used on the surgical side, turned over after skin incision, and the branch of the STA was freed. To protect the deep temporal artery network, the surgeon carefully separated the entire temporalis muscle (in order to avoid vascular pressing, the encephalo-myo-synangiosis part was abandoned if the thickness of the temporalis muscle exceeded 0.8 cm; these patients were excluded from the present study). After removal of the bone flap, the dura was opened in a fan fashion, under the premise of protecting the integrity of the middle meningeal artery. End-to-side anastomosis of the parietal branch of the STA with the M4 branch of the middle cerebral artery (MCA) was performed, followed by extracranial location of the frontal branch of the STA (if the parietal branch was underdeveloped or there was no parietal branch, the frontal branch was used to replace it). After successful anastomosis, intraoperative indocyanine green fluorescence angiography was performed to evaluate graft patency. When the patency of the anastomosis was confirmed, the dura mater was inverted and adhered to the surface of the brain and the temporalis muscle was sutured to the dura at the edge of the bone window. After the bone flap was trimmed (leaving space for the temporal muscle and the graft vasculature to enter the cranial cavity), it was reposited and fixed.

Routine computed tomography (CT) of the head was performed to assess whether there was infarction or bleeding post-operatively. Repeated perfusion CT was performed at 7 days post-surgery to evaluate the improvement in cerebral perfusion.

### 2.4. Statistical analysis

Continuous data are presented as mean ± standard deviation (SD), and the Student's independent *t*-test was used to compare the mean differences between the well-compensated (W) and poorly-compensated (P) groups. To further investigate the association between the index (diameter, flow, PI, RI) and DSA level (W vs. P), a logistic regression using a generalized estimating equation (GEE) model was used. The results for all patients were measured at five time points: baseline (Pre), day 1 (D1), day 7 (D7), month 3 (M3), and month 6 (M6). The change (difference) between adjacent time points for each index was also used in the analysis, including D1 - Pre, D7 - D1, M3 - D7, and M6 - M2. An Autoregressive Process (1) correlation matrix was adopted for the repeated-measure data. A logistic regression coefficient odds ratio (OR) was calculated. A result of *p* < 0.05 would be recognized significant in each two-tailed test. All analyses were performed using SPSS version 25 (SPSS Statistics V25, IBM Corporation, Armonk, New, USA).

## 3. Results

From September 2017 to June 2021, a total of 472 patients with Moyamoya disease were admitted to the Department of Neurosurgery of Nanfang Hospital, 72 of whom did not receive combined bypass, and 349 patients had incomplete post-operative ultrasound data (307 patients lacked complete ultrasound follow-up data; 42 had no 6-month DSA review). Finally, 52 patients (male/female = 25/26) aged 10–58 years (mean, 39.9 ± 14.3 years) with 54 surgically treated hemispheres were included in this study. Baseline demographic data are shown in Table 1. Among them, there were 3 cases in pre-operative Suzuki stage 2, 13 cases in stage 3, 22 cases in stage 4, 14 cases in stage 5, and 2 in stage 6; the admission mRS score was 1 in 23 cases, 2 in 19, 3 in 7, and 4 in 5. There were no statistical differences in baseline data between the sample of patients included in the study and all admitted patients. The demographic data are shown in Supplementary Table 1. A total of 30 hemispheres were grouped into the W group and 24 into the P group according to the DSA examination results. Post-operative CT showed no new hemorrhage or infarction, and CT perfusion imaging showed that cerebral blood flow (CBF) and mean transit time (MTT) significantly improved after surgery. The patients' average discharge mRS score was 0. Except the age composition, there were no statistical differences in age, sex, and bypass side between the groups.

**Table 1:** Comparisons of the demographics and clinical characteristics of the well-compensated collateral group (W group) and poorly-compensated collateral group (P group).

**Parameters**	**Group**		***P*-value**
Case number	28	24	
Hemisphere number	30	24	
Age, years	32.6 ± 13.0	50.6 ± 7.7	0.000
**Gender**			0.169
Male	15 (54%)	10 (42%)	
Female	13 (46%)	14 (58%)	
**Bypass side**			0.207
Left	20 (67%)	11 (46%)	
Right	10 (33%)	13 (54%)	
**Suzuki stage**			0.639
2	2 (7%)	1 (4%)	
3	7 (23%)	6 (25%)	
4	13 (44%)	9 (38%)	
5	7 (23%)	7 (29%)	
6	1 (3%)	1 (4%)	
**Admission mRS**			0.883
1	14 (46%)	9 (38%)	
2	8 (27%)	11 (46%)	
3	5 (17%)	2 (8%)	
4	3 (10%)	2 (8%)	

**Figure 1:**
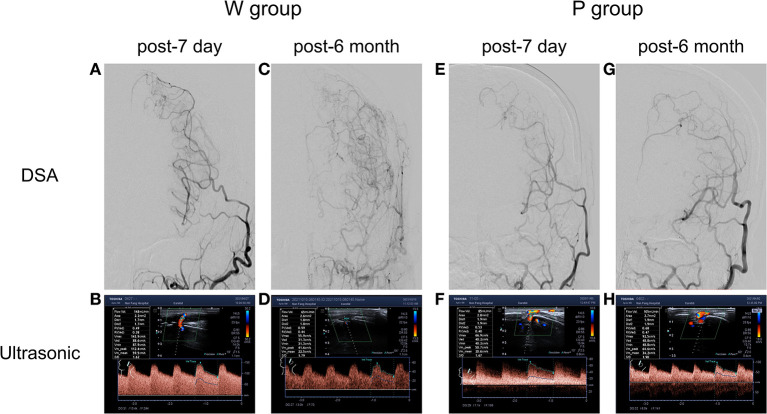
Typical STA grafts with different groups showed at post-operate 7 day and post-operate 6 month. In well-compensated collateral group (W group), **(A)** post-operative 7 day DSA showed the patency of grafts, **(B)** with a high flow rate of 148 ml/min in the ultrasonography examination. **(C)** Post-operative 6 month DSA confirmed a well-compensated collateral, **(D)** with a flow rate of 65 ml/min in the ultrasonography examination. In poorly-compensated collateral group (P group), **(E)** post-operative 7 day DSA showed the patency of grafts, **(F)** with a high flow rate of 85 ml/min in the ultrasonography examination. **(G)** Post-operative DSA confirmed a poorly-compensated collateral, **(H)** with a flow rate of 105 ml/min in the ultrasonography examination.

The post-operative angiographic and ultrasonographic patterns in two representative cases are shown in Figure 1. The first patient (W group) had a low-resistance flow pattern in the STA on ultrasonography, while the second patient (P group) showed a highly resistant flow pattern.

**Figure 2:**
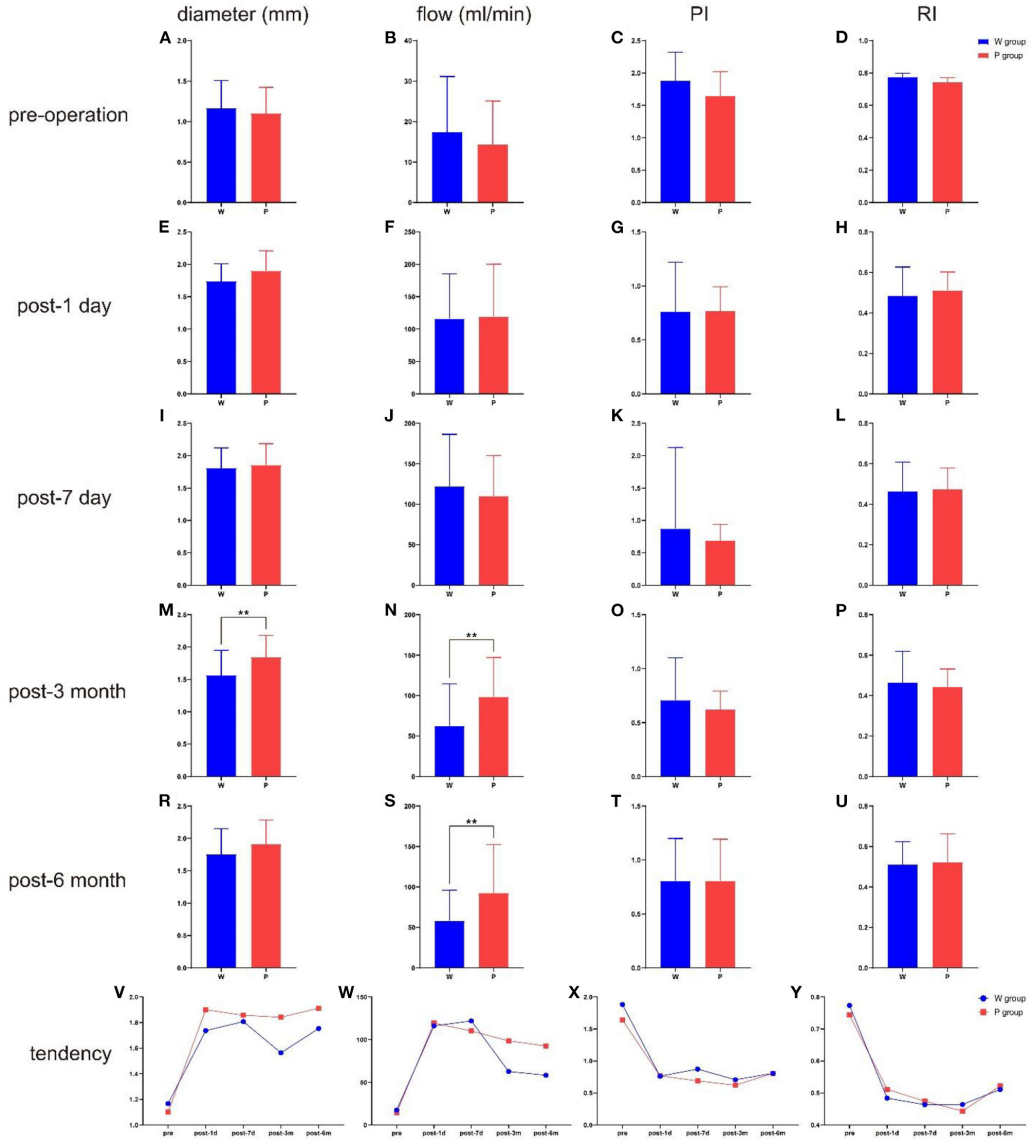
The comparison of ultrasonographic parameters between the two groups in different time point, and the total tendency of different hemodynamics indicators. **(A)** The diameter at pre-operation. **(B)** The flow at pre-operation. **(C)** The PI at pre-operation. **(D)** The RI at pre-operation. **(E)** The diameter at post-operative 1 day. **(F)** The flow at post-operative 1 day. **(G)** The PI at post-operative 1 day. **(H)** The RI at post-operative 1 day. **(I)** The diameter at post-operative 7 day. **(J)** The flow at post-operative 7 day. **(K)** The PI at post-operative 7 day. **(L)** The RI at post-operative 7 day. **(M)** The diameter at post-operative 3 month. **(N)** The flow at post-operative 3 month. **(O)** The PI at post-operative 3 month. **(P)** The RI at post-operative 3 month. **(R)** The diameter at post-operative 6 month. **(S)** The flow at post-operative 6 month. **(T)** The PI at post-operative 6 month. **(U)** The RI at post-operative 6 month. **(V)** The total tendency of diameter. **(W)** The total tendency of flow. **(X)** The total tendency of PI. **(Y)** The total tendency of RI. **P < 0.01.

The ultrasonographic parameters of STA are listed in Table 2. There were no significant pre-operative differences in STA hemodynamics between the two groups (Figure 2). The same pattern of haemodynamic improvement was observed in both groups at follow-up. Compared with pre-operation, the diameter and flow of the graft increased significantly in the short term (3 months) after surgery, while the RI and PI decreased significantly. Six months after surgery, the flow and mean diameter of the graft decreased, but there were still significant differences compared with those before surgery. In the short-term (3 months) after the operation were significant differences in the flow and diameter observed between the two groups. Compared with W group, the P group maintained higher flow and wider diameter at 3 months post-operatively (*P* = 0.012/0.008). This discrepancy in flow even persisted for 6 months or more (*P* = 0.013). However, no statistical differences in RI and PI were found during the follow-up.

**Table 2:** Comparisons of the ultrasonic hemodynamic indicators in different time point of the well-compensated collateral group (W group) and poorly-compensated collateral group (P group).

**Parameters**	**Group**		***P-*value**
**Pre-operative**			
Diameter (mm)	1.17 ± 0.34	1.07 ± 0.29	0.280
Flow (ml/min)	17.4 ± 13.8	13.3 ± 9.5	0.228
PI	1.88 ± 0.44	1.61 ± 0.36	0.022
RI	0.77 ± 0.06	0.74 ± 0.09	0.077
**Post 1 day**			
Diameter (mm)	1.74 ± 0.27	1.89 ± 0.31	0.063
Flow (ml/min)	116.0 ± 69.2	115.1 ± 80.2	0.966
PI	0.76 ± 0.46	0.78 ± 0.22	0.879
RI	0.48 ± 0.14	0.52 ± 0.09	0.350
**Post 7 day**			
Diameter (mm)	1.81 ± 0.31	1.85 ± 0.33	0.656
Flow (ml/min)	121.9 ± 64.5	106.1 ± 47.8	0.360
PI	0.87 ± 1.25	0.70 ± 0.25	0.537
RI	0.46 ± 0.16	0.48 ± 0.11	0.708
**Post 3 month**			
Diameter (mm)	1.56 ± 0.39	1.83 ± 0.35	0.010
Flow (ml/min)	62.6 ± 51.8	97.5 ± 49.3	0.017
PI	0.71 ± 0.39	0.62 ± 0.17	0.339
RI	0.46 ± 0.16	0.44 ± 0.09	0.583
**Post 6 month**			
Diameter (mm)	1.75 ± 0.40	1.90 ± 0.37	0.167
Flow (ml/min)	58.3 ± 37.9	92.9 ± 61.1	0.014
PI	0.81 ± 0.39	0.82 ± 0.39	0.880
RI	0.51 ± 0.11	0.53 ± 0.14	0.578

To explore the effects of different haemodynamic parameters on post-operative revascularization in patients, we performed a GEE logistic regression evaluation (Table 3). Significant estimated ORs were only found in the index flow (*P* = 0.039); the results showed that patients with higher levels of flow were more likely to be in the P group.

**Table 3:** GEE analysis of relationships between well-compensated collateral group (W group) and poorly-compensated collateral group (P group) at post-operative.

**Parameters**	**OR**	**95%CI**	***P***
Diameter (mm)	1.00001	0.99998–1.00005	0.523
Flow (ml/min)	1.0000001	1.000000007–1.0000003	0.039
PI	0.999996	0.99998–1.000009	0.543
RI	1.00001	0.99995–1.00008	0.686

**Figure 3:**
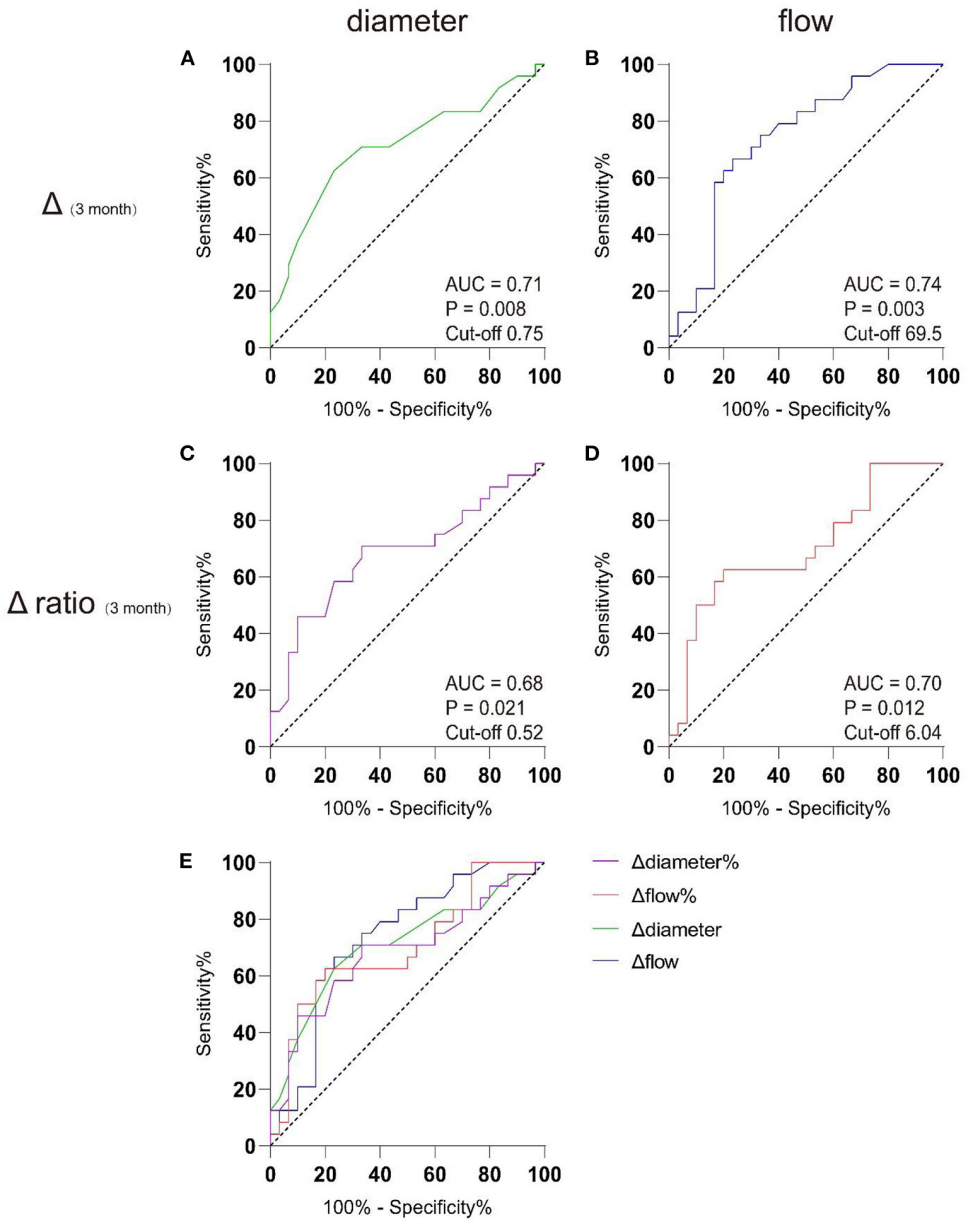
AUC analysis of receiver-operating characteristics. Preoperative data were used as baseline to discriminating well-compensated collateral group (W group) from poor-compensated collateral group (P group) in post-operative 3 month by receiver operating characteristic (ROC) analysis. **(A)** The cut-off value for the widened diameter of the STA graft was 0.75 mm, with the AUC of 0.71. **(B)** The cut-off value for the increased flow of the STA graft was 69.5 ml/min, with area under curve (AUC) of 0.74; **(C)** The cut-off value for the widened diameter ratio of the STA graft was 52%, with the AUC of 0.68; **(D)** The cut-off value for the increased flow ratio of the STA graft was 604%, with area under curve (AUC) of 0.70. **(E)** The comparison between the 4 models.

Based on this finding, we set pre-operative haemodynamic parameters as baseline and attempted to determine cut-off points for parameter changes corresponding to the P group using receiver operating characteristic (ROC) analysis. Table 4 shows the sensitivity, specificity, Youden's index, and cut-off values of ultrasonographic parameters in the prediction of the P group by the Δ1 day (changes between preoperative and post-operative 1 day), Δ7 day, Δ3 month, and Δ6 month, as well as Δ1 day ratio (changes ratio between preoperative and post-operative 1 day), Δ7 day ratio, Δ3 month ratio, and Δ6 month ratio in the STA. Of note, an increase in STA blood flow of 69.5 ml/min or 604% at 3 months after surgery indicated a high risk of poor post-operative collateral compensation. Moreover, if the diameter of the STA increased by 0.75 mm or 52% at 3 months after surgery, this indicated a high risk of poor post-operative collateral compensation. Figure 3 shows the results of the ROC analysis for the prediction of the P group, and the details of each parameter are also shown.

**Table 4:** Pre-operative hemodynamic parameters as the baseline to determine cut-off points for parameter changes corresponding to poorly-compensated collateral group (P group) by receiver operating characteristic (ROC) analysis.

**Parameters**	**AUC (95% CI)**	***P*-value**	**Sensitivity**	**Specificity**	**Youden's index**	**Cut-off**
**1d-pre**						
D	0.61 (0.45–0.77)	0.159				
flow	0.49 (0.33–0.64)	0.855				
PI	0.66 (0.50–0.82)	0.064				
RI	0.68 (0.52–0.83)	0.038	0.61	0.75	0.36	−0.26
**1d-pre ratio**						
D	0.59 (0.43–0.75)	0.081				
flow	0.53 (0.38–0.69)	0.676				
PI	0.66 (0.50–0.82)	0.057				
RI	0.67 (0.51–0.82)	0.052				
**7d-pre**						
D	0.54 (0.36–0.71)	0.695				
flow	0.44 (0.28–0.61)	0.519				
PI	0.64 (0.47–0.80)	0.117				
RI	0.65 (0.49–0.82)	0.087				
**7d-pre ratio**						
D	0.53 (0.36–0.71)	0.715				
flow	0.53 (0.36–0.70)	0.739				
PI	0.63 (0.46–0.80)	0.144				
RI	0.63 (0.46–0.80)	0.144				
**3m-pre**						
D	0.71 (0.57–0.86)	0.008	0.63	0.87	0.39	0.75
flow	0.74 (0.60–0.87)	0.003	0.67	0.87	0.43	69.5
PI	0.59 (0.43–0.75)	0.294				
RI	0.55 (0.39–0.72)	0.522				
**3m-pre ratio**						
D	0.68 (0.54–0.83)	0.021	0.71	0.67	0.38	0.52
flow	0.70 (0.56–0.85)	0.012	0.63	0.80	0.43	6.04
PI	0.57 (0.40–0.73)	0.409				
RI	0.54 (0.37–0.70)	0.655				
**6m-pre**						
D	0.61 (0.46–0.76)	0.166				
flow	0.68 (0.53–0.82)	0.028	0.42	0.90	0.32	91
PI	0.63 (0.48–0.79)	0.111				
RI	0.62 (0.46–0.78)	0.141				
**6m-pre ratio**						
D	0.61 (0.46–0.76)	0.164				
flow	0.65 (0.51–0.80)	0.056				
PI	0.61 (0.45–0.77)	0.187				
RI	0.60 (0.44–0.76)	0.213				

## 4. Discussion

This study showed that the grafts of Moyamoya patients who underwent combined bypass had time-related haemodynamic alterations. These alterations in the STA could suggest different neovascularization patterns in the future. Therefore, we believe that quantitative ultrasound of the STA can be used early in the post-operative period to determine the long-term outcomes of combined bypass surgery in patients with Moyamoya disease. An increase in blood flow ≥69.5 ml/min or 604% and the expansion of diameter ≥0.75 mm or 52% in the post-operative 3 month period, compared with the pre-operative period, suggested poor collateral compensation in the long-term. This may be helpful for clinical interpretation; however, further validation with more cases is needed.

Although we found differences in the age composition in baseline between the two groups, that does not preclude our analysis of the hemodynamics. This age-related phenomenon was considered to conform to clinical experience. Ken Kazumata et al showed that the incidence of postoperative stroke in children with moyamoya disease was significantly lower than in adults (20). Thines et al. also found better Neovascularization rates in children after bypass surgery for moyamoya disease (21). The formation of moyamoya disease and collateral compensation after bypass surgery are generally considered to be associated with increased angiogenesis (22–24). Several factors that promote angiogenesis are thought to be age-related, angiogenic factors are more secreted in the young than in the old (25–27). That makes younger individuals appear to have better revascularization capacity than older patients (28). In addition, hemodynamics is also an important factor in stimulating angiogenesis. The pressure of vessel wall and shear stress of blood flow not only stimulate endothelial migration and promote angiogenesis, but also reflect the state of angiogenesis (29–31). This is the focus of this research.

The changes in the ultrasonographic parameters of STA demonstrated that the flow and diameter of the graft would increase significantly after the operation. The features of the graft's hemodynamics would convert from the original type (high-resistance and low-exhaust) to a compensatory type (low-resistance and high-exhaust) during the post-operation period (32–34). As time progressed, the graft adapted to the growth of collateral compensation, and hemodynamics began to stabilize and gradually recover (35, 36). This is mainly related to the resistance changes in the vascular network distal to the graft (37). The STA originally coursed through the galeal aponeurosis and subcutaneous fascia to supply the scalp and had a typical high-resistance terminal distal vessel. After separation and displacement from under the skin flap, the STA was sutured to the cerebral cortical artery. The distal vascular network of the STA immediately transformed into a low-resistance middle cerebral artery vascular network which suffered long-term ischaemia, and the resistance index dropped significantly (38). In our previous study, at 1 day, 7 days, 3 months, and 6 months after STA-MCA direct bypass surgery, the flow graft was 106.7, 112.6, 97.4, and 79.7 ml/min, respectively (19). This was consistent with the overall alteration trend of graft flow after combined bypass in this study in the well-compensated collateral group (W group), and the poorly-compensated collateral group (P group). This suggests that haemodynamic changes after combined bypass surgery are primarily caused by the direct bypass segment (39).

In this study, a subgroup analysis was performed according to the results of DSA angiography review 6 months after surgery. There was no significant difference in the hemodynamics of the graft between the well-compensated collateral group (W group) and the poorly-compensated collateral group (P group) in the short-term post-operative period. However, significant differences in STA flow and diameter were observed between the two groups at 3 months post-operation, which is thought to be due to the indirect bypass segment (40–42). Indirect revascularization procedures induce neovasculogenesis in the leptomeningeal vascular system by placing highly vascularised tissues, such as vessels, dura, temporalis muscles, periosteum, or galea, on the brain surface. Thus, transdural and transpial collateral establishment is a key point that should be evaluated in patients with Moyamoya disease after indirect bypass surgery (18, 39). In an imaging follow-up study conducted by Houkin et al., MRA showed that the deep temporal artery and middle meningeal artery were significantly thickened to form collateral circulation to compensate intracranially at 3 months after Moyamoya combined bypass surgery (43–45). Charbel et al. conducted a quantitative study on MRA in patients with Moyamoya disease after combined bypass surgery and found a complementary relationship between revascularization of the direct and indirect bypass parts (8). The graft flow showed a significant 69% decrease at the 6-month post-operative time point, and this downward trend appeared 2–3 months post-operatively. After the formation of collateral circulation, the ischaemia of the patients in the well-compensated collateral group (W group) improved gradually, and the burden of the graft decreased. Therefore, compared with the poorly-compensated collateral group without indirect compensation, there was a gradual decline in haemodynamic trends, a decrease in the flow and diameter, and an increase in the resistance and pulsatility index (2, 42, 46). This is consistent with the dynamic complementarity between the direct and indirect parts of the combined bypass surgery, as suggested by previous studies (8, 39). Using ROC analysis with pre-operative parameters as baseline, we found that if STA blood flow was still higher than the pre-operative 69.5 ml/min or 604% and diameter was wider than the pre-operative 0.75 mm or 52% at 3 months post-operatively, the risk of poor post-operative compensation was high. This confirms the role of indirect bypass in joint bypass, but also provides a possibility for us to use ultrasound to evaluate the long-term effect of the operation in the early stage.

To date, DSA angiography remains the gold standard for the diagnosis and review of cerebrovascular disease; however, there is a 0.1–0.3% risk of permanent complications (47–49). During the examinations, patients experience pain and require expensive bed rest after surgery. This makes DSA difficult to perform in an outpatient clinic. Although non-enhanced Timeflight-MRA is a non-invasive method, it still has the disadvantages of being time-consuming and expensive, and it cannot be used for patients with metals, pacemaker implants, and claustrophobia (50–52). Compared with the former two methods, quantitative ultrasonography is a non-invasive, repeatable, outpatient imaging follow-up method (19, 38). The patient examination process is comfortable, inexpensive, and suitable for multiple long-term examinations. The present study used quantitative ultrasonography imaging to show a significant correlation between ultrasonic haemodynamic parameters and the conditions of compensated collaterals in patients with Moyamoya disease after combined bypass surgery. Quantitative ultrasonography not only provides a good description of haemodynamic changes but also predicts collateral compensation at 6 months post-operation in the early period (3 months post-operatively). Therefore, we believe that quantitative ultrasonography may be a useful tool for follow-up of patients with Moyamoya disease after indirect revascularization surgery. We also proposed cut-off points for post-operative change values and the rate of change of flow and diameter in the STA. This is helpful for the early prediction and intervention of poor collateral circulation compensation after combined bypass surgery.

This study had several limitations. First, the number of patients included in the analysis was low. Due to geographical factors, it is difficult for patients who are far away to return to the hospital to complete follow-up according to the doctor. All the examinations and operations were performed in our hospital, which made the selection of patients biased. It is difficult to accurately assess collateral circulation formed after indirect bypass using ultrasonography. It cannot directly quantify the amount of neovascularization, and it is difficult to assess the blood flow of the middle meningeal artery, which is deep and has a large anatomical location. Intra-observer variation is a potential problem in ultrasound examinations.

## 5. Conclusions

Ultrasonographic findings of the STA correlated well with the extent of neovascularization. After combined bypass surgery in patients with Moyamoya disease, the hemodynamics of the STA changed significantly in a short time. Along with the adaptation and compensation of the graft, the blood flow will be lower and the diameter will be thinner at 3 months post-operation. This may be the beginning of angiogenesis and the key timepoint to promote the establishment of collaterals. For patients with ultrasonographic findings indicating insufficient drop in flow and coarctation in diameter, interventions could be considered early.

## Data availability statement

The raw data supporting the conclusions of this article will be made available by the authors, without undue reservation.

## Ethics statement

The studies involving human participants were reviewed and approved by Nanfang Hospital Ethics Committee of Southern Medical University. Written informed consent to participate in this study was provided by the participants' legal guardian/next of kin.

## Author contributions

SC conducted most of the data collection, assembled the results, analyzed data, and wrote the manuscript. BW performed ultrasonic examinations and data analysis. YW, ZW, TL, and JC performed data collection. GZ, ML, SZ, and SQ performed clinical treatment and DSA examinations. GW, JP, and WF provide funds and ideas. All authors contributed to the article and approved the submitted version.

## References

[1] Nguyen VN Motiwala M Elarjani T Moore KA Miller LE Barats M. Direct, indirect, and combined extracranial-to-intracranial bypass for adult Moyamoya disease: an updated systematic review and meta-analysis. Stroke. (2022) 53:3572–82. 10.1161/STROKEAHA.122.039584 36134563

[2] Sun J Li Z-Y Chen C Ling C Li H Wang H. Postoperative neovascularization, cerebral hemodynamics, and clinical prognosis between combined and indirect bypass revascularization procedures in hemorrhagic Moyamoya disease. Clin Neurol Neurosurg. (2021) 208:106869. 10.1016/j.clineuro.2021.106869 34419781

[3] Zhang M Raynald Zhang D Liu X Wang R Zhang Y. Combined STA-MCA bypass and encephalodurosynangiosis versus encephalodurosynangiosis alone in adult hemorrhagic Moyamoya disease: a 5 -year outcome study. J Stroke Cerebrovasc Dis. (2020) 29:104811. 10.1016/j.jstrokecerebrovasdis.2020.104811 32312630

[4] Zhao Y Yu S Lu J Yu L Li J Zhang Y. Direct bypass surgery vs. combined bypass surgery for hemorrhagic Moyamoya disease: a comparison of angiographic outcomes. Front Neurol. (2018) 9:1121. 10.3389/fneur.2018.01121 30619072 PMC6306562

[5] Houkin K Kuroda S Ishikawa T Abe H. Neovascularization (angiogenesis) after revascularization in Moyamoya disease which technique is most useful for Moyamoya disease? Acta Neurochir. (2000) 142:269–76. 10.1007/s007010050035 10819257

[6] Ishii Y Tanaka Y Momose T Yamashina M Sato A Wakabayashi S. Chronologic evaluation of cerebral hemodynamics by dynamic susceptibility contrast magnetic resonance imaging after indirect bypass surgery for Moyamoya disease. World Neurosurg. (2017) 108:427–35. 10.1016/j.wneu.2017.09.001 28893695

[7] Li Y Hu J-W He X-C Cao Y Yu X-B Fu X-J. Bloody fluids located between the temporal muscle and targeted cerebral cortex affect the establishment of indirect collaterals in Moyamoya disease with surgical bypass: a case-control study. Front Neurol. (2022) 13:960199. 10.3389/fneur.2022.960199 36388183 PMC9644190

[8] Amin-Hanjani S Singh A Rifai H Thulborn KR Alaraj A Aletich V. Combined direct and indirect bypass for Moyamoya: quantitative assessment of direct bypass flow over time. Neurosurgery. (2013) 73:962–7. 10.1227/NEU.0000000000000139 23949274

[9] Khan NR Elarjani T Jamshidi AM Lu VM Silva MA Richardson A. Direct bypass surgery for Moyamoya and steno-occlusive vasculopathy: clinical outcomes, intraoperative blood flow analysis, long-term follow-up, and long-term bypass patency in a single surgeon case series of 162 procedures. World Neurosurg. (2022) 168:e500–17. 10.1016/j.wneu.2022.10.015 36216248

[10] Mesiwala AH Sviri G Fatemi N Britz GW Newell DW. Long-term outcome of superficial temporal artery-middle cerebral artery bypass for patients with Moyamoya disease in the US. Neurosurg Focus. (2008) 24:E15. 10.3171/FOC/2008/24/2/E15 18275291

[11] Hasuo K Tamura S Kudo S Uchino A Carlos R Matsushima T. Moyamoya disease: use of digital subtraction angiography in its diagnosis. Radiology. (1985) 157:107–11. 10.1148/radiology.157.1.3898215 3898215

[12] Chen Y Ma L Yang S Burkhardt J-K Lu J Ye X. Quantitative angiographic hemodynamic evaluation after revascularization surgery for Moyamoya disease. Transl Stroke Res. (2020) 11:871–81. 10.1007/s12975-020-00781-5 32056157 PMC7496042

[13] Zhao Y Lu J Zhang Q Zhang Y Zhang D Wang R. Time course of neoangiogenesis after indirect bypass surgery for Moyamoya disease: comparison of short-term and long-term follow-up angiography. Clin Neuroradiol. (2020) 30:91–9. 10.1007/s00062-018-0748-3 30511151

[14] Guo X Gao L Yu H Chen W Yang Y Jin F. Computed tomographic angiography may be used for assessing the dilatation of the anterior choroidal and posterior communicating arteries in patients with Moyamoya syndrome. Eur Radiol. (2021) 31:5544–51. 10.1007/s00330-021-07722-2 33564956

[15] Tian B Jiang Y Kang Q Xu B Liu R Liu Q. Comparative study of 4D CTA and DSA for vascular assessment in Moyamoya disease. Clin Imaging. (2018) 48:74–8. 10.1016/j.clinimag.2017.10.005 29055274

[16] Matsushige T Kraemer M Sato T Berlit P Forsting M Ladd ME. Visualization and classification of deeply seated collateral networks in Moyamoya angiopathy with 7T MRI. AJNR Am J Neuroradiol. (2018) 39:1248–54. 10.3174/ajnr.A5700 29880473 PMC7655433

[17] Shi Z Ma G Zhang D. Haemodynamic analysis of adult patients with Moyamoya disease: CT perfusion and DSA gradings. Stroke Vasc Neurol. (2021) 6:41–7. 10.1136/svn-2019-000317 32883875 PMC8005907

[18] Yeh SJ Tang SC Tsai LK Lee CW Chen YF Liu HM. Color doppler ultrasonography as an alternative tool for postoperative evaluation of collaterals after indirect revascularization surgery in Moyamoya disease. PLoS ONE. (2017) 12:e0188948. 10.1371/journal.pone.0188948 29220356 PMC5722285

[19] Wang G Zhang X Wang B Wen Y Chen S Liu J. Flow evaluation of STA–MCA bypass using quantitative ultrasonography: an alternative to standard angiography for follow up of bypass graft. J Stroke Cerebrovasc Dis. (2020) 29:105000. 10.1016/j.jstrokecerebrovasdis.2020.105000 32807419

[20] Kazumata K Ito M Tokairin K Ito Y Houkin K Nakayama N. The frequency of postoperative stroke in Moyamoya disease following combined revascularization: a single-university series and systematic review. J Neurosurg. (2014) 121:432–40. 10.3171/2014.1.JNS13946 24605834

[21] Thines L Petyt G Aguettaz P Bodenant M Himpens F-X Lenci H. Surgical management of Moyamoya disease and syndrome: current concepts and personal experience. Rev Neurol. (2015) 171:31–44. 10.1016/j.neurol.2014.08.007 25555850

[22] Dorschel KB Wanebo JE. Genetic and proteomic contributions to the pathophysiology of Moyamoya angiopathy and related vascular diseases. Appl Clin Genet. (2021) 14:145–71. 10.2147/TACG.S252736 33776470 PMC7987310

[23] Bang OY Fujimura M Kim SK. The pathophysiology of Moyamoya disease: an update. J Stroke. (2016) 18:12–20. 10.5853/jos.2015.01760 26846756 PMC4747070

[24] Shear A Nishihiro S Hishikawa T Hiramatsu M Sugiu K Yasuhara T. Cerebral circulation improves with indirect bypass surgery combined with gene therapy. Brain Circ. (2019) 5:119–23. 10.4103/bc.bc_33_19 31620658 PMC6785951

[25] Markiewicz M Richard E Marks N Ludwicka-Bradley A. Impact of endothelial microparticles on coagulation, inflammation, and angiogenesis in age-related vascular diseases. J Aging Res. (2013) 2013:734509. 10.1155/2013/734509 24288612 PMC3830876

[26] Ma Q Reiter RJ Chen Y. Role of melatonin in controlling angiogenesis under physiological and pathological conditions. Angiogenesis. (2020) 23:91–104. 10.1007/s10456-019-09689-7 31650428

[27] Watson N Al-Samkari H. Thrombotic and bleeding risk of angiogenesis inhibitors in patients with and without malignancy. J Thromb Haemost. (2021) 19:1852–63. 10.1111/jth.15354 33928747

[28] Scott RM Smith ER. Moyamoya disease and Moyamoya syndrome. N Engl J Med. (2009) 360:1226–37. 10.1056/NEJMra0804622 19297575

[29] Kiefer FN Neysari S Humar R Li W Munk VC Battegay EJ. Hypertension and angiogenesis. Curr Pharm Des. (2003) 9:1733–44. 10.2174/1381612033454540 12871205

[30] Elieh AKD Wöhrl S Bielory L. Mast cell biology at molecular level: a comprehensive review. Clin Rev Allergy Immunol. (2020) 58:342–65. 10.1007/s12016-019-08769-2 31828527

[31] Li M Kroetz DL. Bevacizumab-induced hypertension: clinical presentation and molecular understanding. Pharmacol Ther. (2018) 182:152–60. 10.1016/j.pharmthera.2017.08.012 28882537 PMC5785435

[32] Kim H Jang D-K Han Y-M Sung JH Park IS Lee K-S. Direct bypass versus indirect bypass in adult Moyamoya angiopathy with symptoms or hemodynamic instability: a meta-analysis of comparative studies. World Neurosurg. (2016) 94:273–84. 10.1016/j.wneu.2016.07.009 27423200

[33] Fujimura M Tominaga T. Flow-augmentation bypass for Moyamoya disease. J Neurosurg Sci. (2021) 65:277–86. 10.23736/S0390-5616.20.05079-1 33245218

[34] Yu J Shi L Guo Y Xu B Xu K. Progress on complications of direct bypass for Moyamoya disease. Int J Med Sci. (2016) 13:578–87. 10.7150/ijms.15390 27499690 PMC4974906

[35] Kim T Bang JS Kwon O-K Hwang G Kim JE Kang H-S. Hemodynamic changes after unilateral revascularization for Moyamoya disease: serial assessment by quantitative magnetic resonance angiography. Neurosurgery. (2017) 81:111–9. 10.1093/neuros/nyw035 28327889

[36] Zhang K Ren W Sun Y-X Wang X-J Li C-Y Wang Z-L. Angiographic characteristics of cerebral perfusion and hemodynamics of the bridging artery after surgical treatment of unilateral Moyamoya disease. Front Neurosci. (2022) 16:922482. 10.3389/fnins.2022.922482 35774553 PMC9239480

[37] Zhu F-P Zhang Y Higurashi M Xu B Gu Y-X Mao Y. Haemodynamic analysis of vessel remodelling in STA-MCA bypass for Moyamoya disease and its impact on bypass patency. J Biomech. (2014) 47:1800–5. 10.1016/j.jbiomech.2014.03.032 24720886

[38] Kraemer M Schuknecht B Jetzer AK Yonekawa Y Khan N. Postoperative changes in the superficial temporal artery and the external carotid artery duplex sonography after extra-intracranial bypass surgery in European Moyamoya disease. Clin Neurol Neurosurg. (2012) 114:930–4. 10.1016/j.clineuro.2012.02.004 22480619

[39] Komura S Mikami T Sugino T Suzuki Y Komatsu K Wanibuchi M. Complementary relation between direct and indirect bypass in progress of collateral circulation in Moyamoya disease. World Neurosurg. (2017) 104:197–204. 10.1016/j.wneu.2017.04.122 28478244

[40] Kuroda S Houkin K Ishikawa T Nakayama N Iwasaki Y. Novel bypass surgery for Moyamoya disease using pericranial flap: its impacts on cerebral hemodynamics and long-term outcome. Neurosurgery. (2010) 66:1093–101. 10.1227/01.NEU.0000369606.00861.91 20495424

[41] Takahashi JC Funaki T Houkin K Kuroda S Fujimura M Tomata Y. Impact of cortical hemodynamic failure on both subsequent hemorrhagic stroke and effect of bypass surgery in hemorrhagic Moyamoya disease: a supplementary analysis of the Japan adult Moyamoya trial. J Neurosurg. (2020) 134:940–5. 10.3171/2020.1.JNS192392 32168484

[42] Uchino H Kim J-H Fujima N Kazumata K Ito M Nakayama N. Synergistic interactions between direct and indirect bypasses in combined procedures: the significance of indirect bypasses in Moyamoya disease. Neurosurgery. (2017) 80:201–9. 10.1227/NEU.0000000000001201 28175916

[43] Houkin K Aoki T Takahashi A Abe H. Diagnosis of Moyamoya disease with magnetic resonance angiography. Stroke. (1994) 25:2159–64. 10.1161/01.STR.25.11.2159 7974539

[44] Yamamoto T Okada T Fushimi Y Yamamoto A Fujimoto K Okuchi S. Magnetic resonance angiography with compressed sensing: an evaluation of Moyamoya disease. PLoS ONE. (2018) 13:e0189493. 10.1371/journal.pone.0189493 29351284 PMC5774704

[45] Houkin K Nakayama N Kuroda S Ishikawa T Nonaka T. How does angiogenesis develop in pediatric Moyamoya disease after surgery? A prospective study with MR angiography. Childs Nerv Syst. (2004) 20:734–41. 10.1007/s00381-004-0971-x 15197570

[46] Kohno K Oka Y Kohno S Ohta S Kumon Y Sakaki S. Cerebral blood flow measurement as an indicator for an indirect revascularization procedure for adult patients with Moyamoya disease. Neurosurgery. (1998) 42:752–7. 10.1097/00006123-199804000-00043 9574639

[47] Waugh JR Sacharias N. Arteriographic complications in the DSA era. Radiology. (1992) 182:243–6. 10.1148/radiology.182.1.1727290 1727290

[48] van Asch CJJ Velthuis BK Rinkel GJE Algra A de Kort GAP Witkamp TD. Diagnostic yield and accuracy of CT angiography, MR angiography, and digital subtraction angiography for detection of macrovascular causes of intracerebral haemorrhage: prospective, multicentre cohort study. BMJ. (2015) 351:h5762. 10.1136/bmj.h5762 26553142 PMC4637845

[49] Burger IM Murphy KJ Jordan LC Tamargo RJ Gailloud P. Safety of cerebral digital subtraction angiography in children: complication rate analysis in 241 consecutive diagnostic angiograms. Stroke. (2006) 37:2535–9. 10.1161/01.STR.0000239697.56147.77 16946163

[50] Chen Q Qi R Cheng X Zhou C Luo S Ni L. Assessment of extracranial-intracranial bypass in Moyamoya disease using 3T time-of-flight MR angiography: comparison with CT angiography. Vasa. (2014) 43:278–83. 10.1024/0301-1526/a000363 25007906

[51] Miyakoshi A Funaki T Fushimi Y Kikuchi T Kataoka H Yoshida K. Identification of the bleeding point in hemorrhagic Moyamoya disease using fusion images of susceptibility-weighted imaging and time-of-flight MRA. AJNR Am J Neuroradiol. (2019) 40:1674–80. 10.3174/ajnr.A6207 31515213 PMC7028580

[52] Fürst G Saleh A Wenserski F Malms J Cohnen M Aulich A. Reliability and validity of noninvasive imaging of internal carotid artery pseudo-occlusion. Stroke. (1999) 30:1444–9. 10.1161/01.STR.30.7.1444 10390321

