# Supplementary material for: Ultrasonic hemodynamic changes of superficial temporal artery graft in different angiogenesis outcomes of Moyamoya disease patients treated with combined revascularization surgery
Source: Front Neurol. 2023 Feb 16;14:1115343. doi: 10.3389/fneur.2023.1115343 (PMC9978192; doi:10.3389/fneur.2023.1115343)
Supplement: Supplementary file 1 [file Table_1.docx]

Supplementary Material

Table S1. Demographics and clinical characteristics for patient included and admitted.

| **Parameters** | **included** | **admitted** | **P-value** |
| --- | --- | --- | --- |
| **Case number** | 52 | 472 |  |
| **Hemisphere number** | 54 | 513 |  |
| **Age, years** | 39.9±14.3 | 44.4±13.7 | 0.060 |
| **Gender** |  |  | 0.532 |
| Male | 25 (48%) | 254 (42%) |  |
| Female | 27 (52%) | 218 (58%) |  |
| **Bypass side** |  |  | 0.270 |
| Left | 31 (57%) | 277 (54%) |  |
| Right | 23 (43%) | 236 (46%) |  |
| **Suzuki stage** |  |  | 0.790 |
| 2 | 3 (6%) | 36 (7%) |  |
| 3 | 13 (24%) | 118 (23%) |  |
| 4 | 22 (40%) | 216 (42%) |  |
| 5 | 14 (26%) | 128 (25%) |  |
| 6 | 2 (4%) | 15 (3%) |  |
| **Admission mRS** |  |  | 0.816 |
| 1 | 23 (43%) | 231 (45%) |  |
| 2 | 19 (35%) | 164 (32%) |  |
| 3 | 7 (13%) | 77 (15%) |  |
| 4 | 5 (9%) | 41 (8%) |  |
